# NEK7 interacts with NLRP3 to modulate the pyroptosis in inflammatory bowel disease via NF-κB signaling

**DOI:** 10.1038/s41419-019-2157-1

**Published:** 2019-12-02

**Authors:** Xueliang Chen, Ganglei Liu, Yuanyuan Yuan, Guotao Wu, Shalong Wang, Lianwen Yuan

**Affiliations:** 1 Department of Geriatric Surgery, The Second Xiangya Hospital, Central South University, 410011 Changsha, China; 2 Department of General Surgery, The people’s Hospital of Baoan Shenzhen, 518000 Shenzhen, China

**Keywords:** Cell biology, Diseases

## Abstract

Inflammatory bowel disease (IBD) is one of the most common diseases in the gastrointestinal tract related to aberrant inflammation. Pyroptosis, which is characterized by inflammasome formation, the activation of caspase-1, and the separation of the N- and C-terminals of GSDMD, might be related to IBD pathogenesis. NEK7 is an important component of the NLRP3 inflammasome in macrophages. We attempted to investigate the mechanism of NEK7 interacting with NLRP3 to modulate the pyroptosis in IBD. NEK7 mRNA and protein expression and pyroptosis-associated factors, including Caspase-1 (p45, p20), NLRP3, and GSDMD, were upregulated in IBD tissues. NEK7 knockdown abolish ATP + LPS-induced pyroptosis in vitro and improved DSS-induced chronic colitis in vivo. NEK7 interacted with NLRP3, as revealed by Co-IP and GST pull-down assays, to exert its effects. Moreover, short-term LPS treatment alone induced no significant changes in NEK7 protein level. TLR4/NF-κB signaling in MODE-K cells could be activated by LPS treatment. LPS-induced NEK7 upregulation could be significantly reversed by JSH-23, an inhibitor of p65. Furthermore, LUC and ChIP assays revealed that RELA might activate the transcription of NEK7 via targeting its promoter region. LPS-induced TLR4/NF-κB activation causes an increase in NEK7 expression by RELA binding NEK7 promoter region. In conclusion, NEK7 interacts with NLRP3 to modulate NLRP3 inflammasome activation, therefore modulating the pyroptosis in MODE-K cells and DSS-induced chronic colitis in mice. We provide a novel mechanism of NEK7-NLRP3 interaction affecting IBD via pyroptosis.

## Introduction

Inflammatory bowel disease (IBD) is the most commonly-seen aberrant inflammation-associated disease in the gastrointestinal tract^1,2^. IBD is a complex disorder with genetic, immune system, microbiome, and environmental influences that can be subdivided into two distinct forms: ulcerative colitis and Crohn’s disease, which are associated with dysregulated inflammation either restricted to the colon or throughout the gastrointestinal tract, respectively. The loss of immune system homeostasis is a hallmark of IBD^3–12^.

As a unique pattern of inflammation-related cell death, pyroptosis is associated with inflammasome formation^13^. The classical inflammasome formation requires signal 1 (transcriptional priming) and signal 2 (ATP); whereas, non-classical inflammasome signaling can proceed without the dual signals and was defined in the context of the NOD-like receptor pyrin domain-containing protein 3 (NLRP3) inflammasome following LPS exposure^14^. Following LPS-induced activation of TLR4/NF-κB, gasdermin D (GSDMD), which was identified by two independent screening approaches as a key effector of pyroptosis^15^, could be transcriptionally activated by NF-κB^16^. When extracellular signals associated with pyroptosis activate inflammasomes, such as NLRP3 inflammasomes containing core unit of the inflammasome like NLRP3, ASC and caspase-1, they subsequently cleave and activate caspases-1, -4, -5, and -11. Consequently, activated caspase-1 cleaves and separates the N- and C-terminals of GSDMD^14^. As a result, the N-terminal fragment of GSDMD constitutes nanopores in the cell membrane, resulting in cell swelling, the cleavage of pro-IL-1β and pro-IL-18 into mature forms, as well as the cell death related to inflammation, namely pyroptosis^17–20^. Based on the above-mentioned findings, the potential of IBD treatment via binding to cell death signaling pathways could be crucial.

It has been shown by previous positive genetic analysis on the activation of inflammasome in C57BL/6J mice that one of 11 NEK kinases discovered in vertebrates, namely NIMA-related kinase 7 (NEK7), is an important component of the NLRP3 inflammasome in macrophages^21^. NEK7 is identified as a highly conserved serine or threonine kinase, which is not only crucial for mitosis entry, cell cycle progression, cell division, and mitotic process, but also expressed in brain, heart, lung, liver, spleen, and other issues. The lower activity of NEK7 within natural growth conditions could be of importance for maintaining homeostasis. Nevertheless, any disruption with homeostasis could be accompanied by the dysregulation of NEK7, leading to the growth of abnormal cells, such as the multinuclear cells and apoptotic cells strongly related to inflammation^22–24^. In terms of regulating the activation of NLRP3 inflammasome, we discovered that NLRP3 gets a leucine-rich repeat domain, to which NEK7 is easy to bind. Moreover, it has been revealed by He et al.^21^ that as the downstream of potassium efflux, NEK7 is involved in NLRP3 activation process. Furthermore, it has been found that potassium efflux could be interrupted by 50 mM KCl to disturb the interaction between NEK7 and NLRP3 caused through the stimulation of NLRP3 activation, resulting in the inhibition to formation of NLRP3 complex. Besides, mutant NLRP3 need NEK7 to activate Caspase-1 without potassium efflux. In conclusion, inflammasome activation of NEK7 is closely related to that of NLRP3. Thus, it is reasonable to hypothesize that NEK7 may interact with NLRP3 to modulate the inflammasome activation and subsequent pyroptosis, finally affecting IBD progression.

Herein, NEK7 mRNA and protein expression and pyroptosis-related factors, including Caspase-1 (p45, p20), NLRP3, and GSDMD, were first examined. In vitro inflammasome stimulation and cellular pyroptosis model was conducted in intestinal epithelial cell line, MODE-K, by ATP followed by LPS stimulations. In vivo chronic DSS-induced colitis model was constructed in mice by DSS induction. Afterward, NEK7 knockdown was achieved in vitro and in vivo for evaluation of NEK7 effect on cell pyroptosis and IBD process. As for the molecular mechanism, the interaction between NEK7 and NLRP3 was validated through co-immunoprecipitation (Co-IP) and GST pull-down assays. Finally, the involvement of LPS-induced TLR4/NF-κB activation was validated by immunoblotting, Luciferase reporter, and chromatin immunoprecipitation (ChIP) assays. In summary, we provide a solid experimental basis for understanding a novel mechanism by which NEK7 interacts with NLRP3 to modulate the inflammasome activation and cell pyroptosis, finally affecting IBD progression.

## Materials and methods

### Clinical tissue samples

A total of 15 cases ulcerative colitis tissues and 15 paired control tissues from distal end of colon ulcers were collected from patients with IBD undergoing resection surgery at the Second Xiangya Hospital after signing informed consents. The whole study was approved by the Ethic Committee of The Second Xiangya Hospital of Central South University (approval number: 2017-S117). Newly diagnosed and untreated patients that had no other inflammatory or infectious disease were included. Post colonoscopies, patients with IBD were included. The physician global assessment (PGA) was used to classify disease activity, based on medical history, physical examination, laboratory findings and endoscopic examination^25^. Upon diagnostic colonoscopy, intestinal biopsies were collected for research purposes. The biopsies were snap frozen in liquid nitrogen. Frozen biopsies were stored at −80 °C until further analyses.

### Cell culture and cell transfection

Mouse intestinal epithelial cell line, MODE-K, was purchased from Shanghai GuanDao Biological Engineering (Shanghai, China) and cultured in Eagle’s Minimum Essential Medium supplemented with 10% FBS (Invitrogen, Carlsbad, CA, USA), 2 mM L-glutamine, 100 IU penicillin, and 100 mg/ml streptomycin at 37 °C in a humidified incubator containing 5% CO_2_. For NEK7 knockdown, specific small interfering RNA (NEK7 siRNA1 and NEK7 siRNA2, GenePharma, Shanghai, China) were transfected into MODE-K cells using lipo2000 (Invitrogen). The scramble siRNA were used as negative control (si-NC). The sequences were listed below: NEK7 siRNA1 sense: GAUAGACUGUGUUUAUAGATT; antisense: UCUAUAAACACAGUCUAUCTT; NEK7 siRNA2 sense: GAAUGAUAAAGCACUUUAATT, antisense: UUAAAGUGCUUUAUCAUUCTT; si-NC sense: UUCUCCGAACGUGUCACGUTT, antisense: ACGUGACACGUUCGGAGAATT.

### Inflammasome stimulation and determination of pyroptotic cell death

For NLRP3 inflammasome stimulation, cells transfected with siRNA were stimulated by 5 mM adenosine 5-triphosphate (ATP) (SunShine Biotechnology, Nanjing, China) for 30 min alone and then with 200 ng/ml lipopolysaccharide (LPS) (Sigma-Aldrich; St. Louis, MO, USA) alone for 4 h prior to pyroptosis detection. The morphology of pyrolytic cells was examined under a light microscopy. Cells were stained with propidium iodide (PI) to mark the membrane pores (Life Technology, Carlsbad, CA, USA).

### Animals

Female C57BL/6 mice aged 6–8 weeks purchased from the SLAC experimental animal center (Changsha, China), and were housed in the animal facilities with a 12:12-h light/dark cycle, controlled temperature (22–24 °C) and humidity (50–60%), for 1-week quarantine with free access to water and food. All procedures were approved by The Animal Care and Use Committee of Central South University.

### Establishment and identify of DSS-induced chronic colitis model in mice

Each mouse received 4 cycles of DSS treatment consisting of 7 days with 1.5% DSS in the drinking water followed by a 10 days recovery phase with normal drinking water. After the last DSS cycle, mice received normal drinking water for 4 weeks. For chronic DSS-induced colitis, the mice were administered 1.5% DSS (molecular weight, 36,000–50,000; MP Biomedicals, Solon, OH, USA) in their drinking water for 7 days followed by a 7-day recovery phase with distilled water. The control group animals were administered distilled water. Each group contains 8 mice. The mice were monitored daily for survival and body weight. For the histological analysis, the large and small intestine tissues were fixed in 4% paraformaldehyde and embedded in paraffin. Fixed tissues were cut into 5-mm-thick sections, placed on glass slides and deparaffinized. The sections were stained with hematoxylin and eosin (H&E) and observed under a light microscope.

For lentivirus shRNA transfection, the lentivirus package system containing NEK7-shRNA or scramble shRNA vector were transfected to HEK293FT cells and Lsh NEK7 and Lsh NC lentiviruss were harvested by PEG precipitation. For in vivo studies, 14 DSS mice were randomly divided into Lsh NC group and Lsh NKE7 group, 100 μl of lentivirus (1 × 10^10^ TU) was delivered by intraperitoneal injection three times (at week 0, 2, and 4 during DSS establishment). H&E staining and body weight determination were performed as above-described.

### Assessment of disease activity index

To assess the severity of colitis, the body weight, stool consistence, and blood in the stool were determined according to previously published grading system^26^. Briefly, weight loss was scored as follows: score 0, none; score 1, 1–5%; score 2, 5–10%; score 3, 10–20%; score 4, >20%. Diarrhea was scored as follows: score 0, normal; score 2, loose stools; score 4, watery diarrhea. Blood in stool was scored as follows: score 0, normal; score 2, slight bleeding; score 4, gross bleeding. These composed the disease activity index (DAI) score for the assessment of disease severity.

### Total RNA extraction and real-time PCR

Total RNA was extracted using Trizol reagent (Invitrogen) following the protocol. The SYBR green PCR Master Mix (Qiagen) was used for mRNA expression detection following the protocol. The β-actin expression was used as an endogenous control. 2^−ΔΔ^CT method was used to analyze the relative fold changes.

### Immunoblotting analyses

The protein levels of NEK7, Caspase-1 (p45 and p20), NLRP3, GSDMD (-FL/-N), TLR4, MyD88, p65 and p-p65 in cells were detected by performing immunoblotting assays using β-actin as an endogenous normalization. Cells were lysed by RIPA buffer (Sigma-Aldrich, USA) with Complete Protease Inhibitor Cocktail (Roche, USA). Cell lysates were transferred to 1.5 mL tube and kept at −20 °C before use. SDS-PAGE was conducted to separate the cellular proteins. Proteins were loaded onto SDS-PAGE minigel, and then transferred onto PVDF membrane. The blots were probed with antibodies at 4 °C overnight and incubated with HRP-conjugated secondary antibody. Signals were visualized using ECL Substrates (Millipore, USA). The protein expression was normalized to endogenous β-actin. All antibodies used were listed in Table S1.

### Enzyme-linked immunosorbent assay

The concentrations of lL-1β in the culture supernatants were determined using commercial mouse lL-1β ELISA kit (ab100704, Abcam, Cambridge, MA, USA) following the manufacturer’s instructions.

### ASC speck staining and ASC oligomer cross-linking

Cells were plated on an 8-well permanox chamber slide (Thermo Scientific, cat no. 177445) overnight. Cells were primed with 200 ng/ml LPS for 4 h and then stimulated with ATP. After stimulation, cells were fixed with 4% paraformaldehyde, permeabilized with 0.1% Triton X-100, and the slides blocked with PBS buffer containing 3% BSA. Cells were stained with anti-ASC antibody and Alexa Fluor 488-conjugated secondary antibody. DAPI was used to stain nuclei. Cell images were taken using an Olympus Fluo-View 500 confocal microscope system.

### GST pull-down assay

The sequences encoding NEK7 were cloned into a pEGX-6P-1 vector which contained open reading frame (ORF) of GST tag. The sequences encoding NLRP3 were cloned into pET22b(+) vector which contained ORF of 6 × His tag. NEK7-GST and NLRP3-His fusion proteins were expressed in *Escherichia coli* BL21 (DE3) and purified as previously described^27^. GST pull-down assay was employed to identify the interactions between NEK7 and NLRP3. Briefly, purified GST-fused proteins were incubated with prepared glutathione sepharose beads (Byotime, Shanghai, China) on the rotating incubator at 4 °C for overnight, and then the beads were collected and washed 3 times. 0.1 mg/mL of input proteins were dissolved in the reaction buffer (20 mM Tris, 100 mM NaCl, 1 mM DTT and 1 mM EDTA) and incubated with the beads on the rotating incubator at 4 °C for 3 h. After removing the supernatant, the beads were washed with the reaction buffer 4 times. The target proteins were eluted and resolved with 10% SDS. These elute were then analyzed and detected by SDS-PAGE and western blotting.

### Co-IP assay

The sequence encoding NEK7 and NLRP3 were cloned into the pcDNA-Flag or pcDNA-Myc vector, named Flag-NEK7 and Myc-NLRP3, respectively. The eukaryotic expression vectors, Flag-NEK7 and Myc-NLRP3, which express NEK7 and NLRP3, respectively, were constructed and co-transfected into MODE-K cells. Empty vectors were co-transfected into target cells as controls. 36 h after transfection, the cells were harvested, and the proteins were extracted. Flag monoclonal antibodies were used for IP testing, followed by Western blot detection using Flag and Myc antibodies. In order to exclude the effect of DNase and RNase, we treated the cell lysates with 5 mg/ml Dnase and Rnase, respectively.

### Luciferase reporter assays for NEK7 transcriptional activity determination

Briefly, p65 response element (p65 RE) and either wild-type or mutated NEK7 luciferase reporter vectors (containing a mutation in any of the predicted p65 binding sites) were transfected into the MODE-K cells. After overnight transfection, cells were then lysed, and luciferase activity was measured with a Promega kit (Promega, Madison, WI) and a microplate reader (Bio-rad, USA).

### Chromatin immunoprecipitation

Briefly, the treated cells were cross-linked with 1% formaldehyde, sheared to an average size of 400 bp DNA, and immunoprecipitated using antibodies against p65. The ChIP-PCR primers were designed to amplify the promoter regions containing putative p65 binding sites within NEK7. A positive control antibody (RNA polymerase II) and a negative control non-immune IgG were used to demonstrate the efficacy of the kit reagents (Epigentek Group, NY, USA, P-2025-48). The immunoprecipitated DNA was subsequently cleaned, released, and eluted. The eluted DNA was used for downstream applications, such as ChIP-PCR. The fold-enrichment (FE) was calculated as the ratio of the amplification efficiency of the ChIP sample to that of the non-immune IgG. The amplification efficiency of RNA Polymerase II was used as a positive control. FE% = 2 (IgG CT-Sample CT) × 100%.

### Statistical analysis

Data are processed using SPSS17.0 statistical software and presented as the mean ± S.D. of results from at least three independent experiments. A Student *t* test (two-tails) was used for statistical comparison between means where applicable. Differences among more than two groups in the above assays were estimated using one-way ANOVA. *P < 0.05; **P < 0.01.

## Results

### Expression and protein levels of NEK7 and pyroptosis-related factors in tissue specimens

**Fig. 1:**
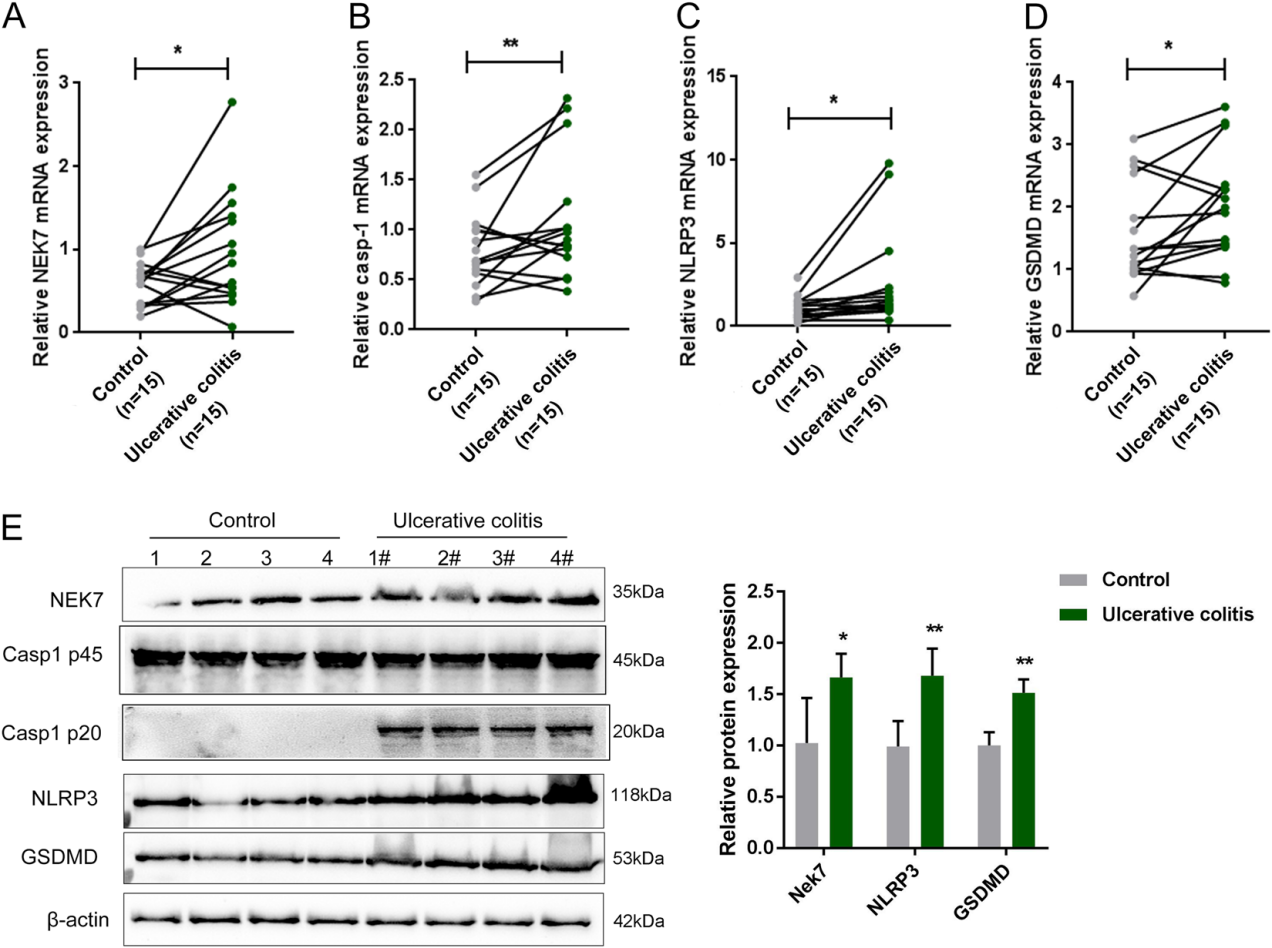
Expression and protein levels of NEK7 and pyroptosis-related factors in tissue samples. **a**–**d** The mRNA expression of NEK7, Caspase-1, NLRP3, and GSDMD in 15 cases of inflammatory bowel disease (ulcerative colitis) and 15 paired control tissues examined by real-time PCR. **e** The protein levels of NEK7, Caspase-1, NLRP3, and GSDMD in 15 cases of ulcerative colitis and 15 paired control tissues examined by Immunoblotting. *P < 0.05, **P < 0.01, compared to normal (non-IBD) group.

For studying how NEK7 affected the pyroptosis in IBD, we first examined the mRNA expression and protein levels of NEK7, as well as key factors in pyroptosis, including Caspase-1, NLRP3, and GSDMD, in control and ulcerative colitis tissues in IBD patients. As shown in Fig. 1a–d, the mRNA expression of NEK7, Caspase-1, NLRP3, and GSDMD showed to be remarkably increased in ulcerative colitis tissue samples than that in control tissues. Consistently, the protein levels of these factors were remarkably increased in ulcerative colitis tissue samples than those in control tissue samples (Fig. 1e).

### Effect of NEK7 on the pyroptosis of intestinal epithelial cell in vitro

To investigate the specific effects of NEK7 on the pyroptosis in vitro, we established cellular pyroptosis model in a mouse intestinal epithelia cell line, MODE-K, by stimulating the cells with 200 ng/ml LPS alone for 4 h, or with 5 mM ATP alone for 30 min and then with 200 ng/ml LPS alone for 4 h. Under light microscopy, the cells became round and swollen under LPS stimulation alone. After ATP + LPS stimulation, the typical appearance of cell pyroptosis (yellow arrow) can be seen: the cells were swollen, the nuclei were concentrated, and vesicle-like pyroptotic bodies were formed (Supplementary Fig. S1A, upper panel). PI staining showed that after short-term stimulation, cells in the mock group were not stained, suggesting no cell pyroptosis existed in mock group (Supplementary Fig. S1A, bottom panel). However, under short-term treatment, the number of PI-positive stained cells within ATP + LPS-stimulated group could be remarkably increased, compared to that in the LPS-stimulated group (Supplementary Fig. S1A, bottom panel), indicating that ATP + LPS co-stimulation could successfully induce a model of pyroptosis in MODE-K cells. Consistently, IF staining also revealed that ASC specks only appeared in ATP + LPS-stimulated group (Supplementary Fig. S1B). Importantly, as shown in Supplementary Fig. S1C, D, the protein levels of Caspase-1 (p20), and GSDMD-N could be only detected after co-stimulation of ATP + LPS, further indicating that pyroptosis could be induced by ATP + LPS stimulation. However, short-term LPS stimulation alone (4 h treatment) caused no significant changes in NEK7 and NLRP3 protein levels (Supplementary Fig. S1C), suggesting the existences of different mechanisms upon short-term LPS stimulation.

**Fig. 2:**
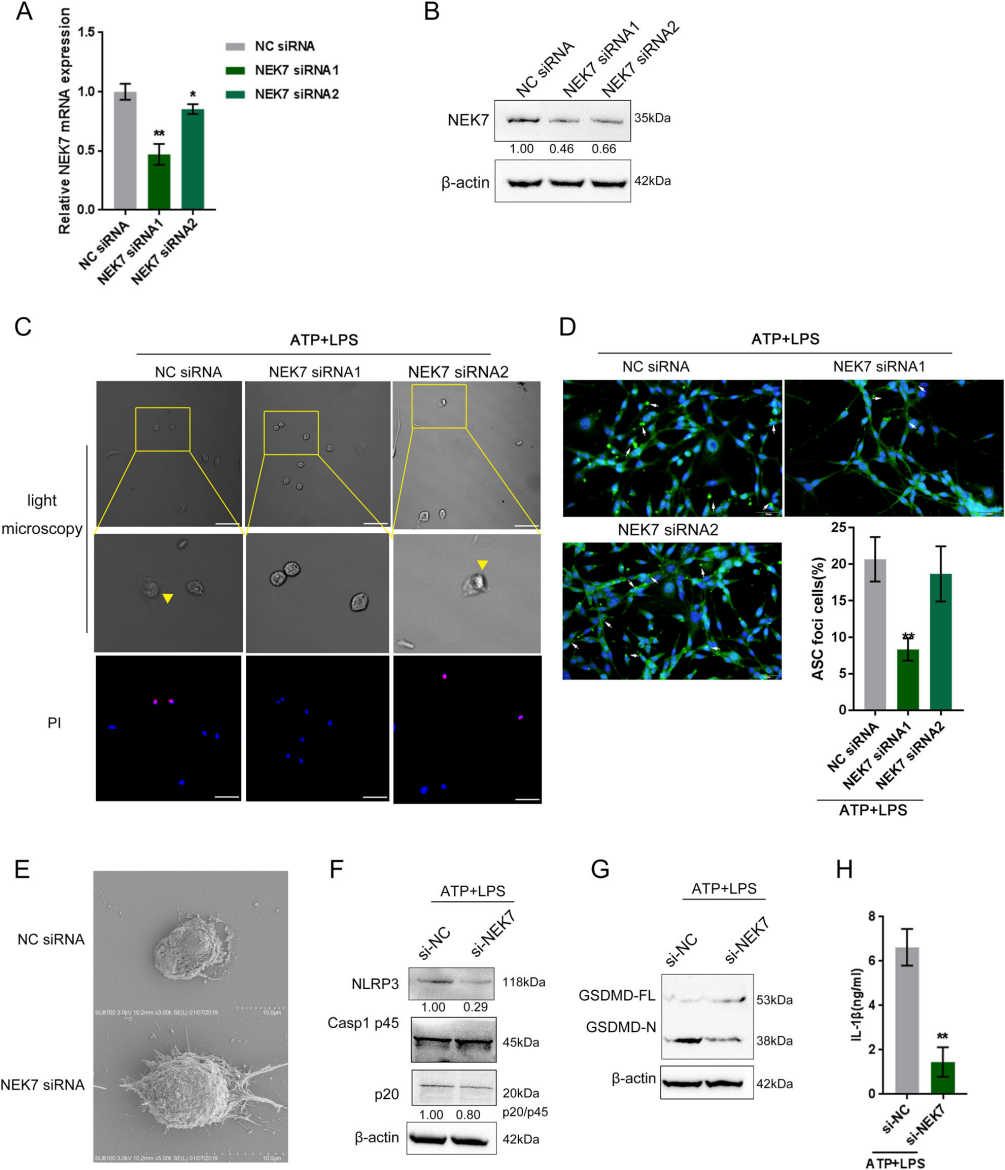
Effect of NEK7 on the pyroptosis of intestinal epithelial cell in vitro. **a**, **b** NEK7 knockdown in intestinal epithelial cell line, MODE-K, was achieved by transfection of NEK7 siRNA1 or siRNA2, as confirmed by real-time PCR and Immunoblotting. **c** MODE-K cells were transfected with NEK7 siRNA1 or siRNA2 and treated with ATP + LPS (5 mM ATP for 30 min then treated with 200 ng/ml LPS for 4 h) for pyroptosis induction and cellular pyroptosis phenotype was examined under light microscopy and using PI staining (scale bar, 50 μm). **d** Immunofluorescence staining and quantitative analysis were performed to detect the formation of the ASC specks (scale bar, 50 μm). **e** The cell morphology of si-NC (negative control)-transfected or si-NEK7-transfected MODE-K cells were observed and imaged under a scanning electron microscope (SEM) (scale bar, 10 μm). **f** The protein levels of NEK7, NLRP3, and Caspase-1 (p45, p20) in si-NC-transfected or si-NEK7-transfected MODE-K cells under ATP + LPS treatment examined by Immunoblotting. **g** The levels of GSDMD-FL and GSDMD-N examined by Immunoblotting. **h** IL-1β content in supernatant determined by ELISA. *P < 0.05, **P < 0.01, compared to si-NC (NC siRNA) group.

Next, we examined the effects of NEK7 on pyroptosis in MODE-K cells. Then we transfected si-NEK7 (siRNA1 and siRNA2) to achieve NEK7 silence, and performed real-time PCR (Fig. 2a) and Immunoblotting to verify the transfection efficiency (Fig. 2b). Once transfected, cells were stimulated by ATP + LPS for pyroptosis induction. Under light microscopy, pyroptotic phenotype could be observed in NC siRNA (negative control)-transfected group and NEK7 siRNA2-transfected group (Fig. 2c). PI staining revealed similar results (Fig. 2c). As a further confirmation, IF staining revealed that the percentage of ASC specks was significantly reduced in NEK7 siRNA1-transfected group (Fig. 2d). These data indicate that NEK7 knockdown could abolish ATP + LPS-induced pyroptosis in MODE-K cells; NEK7 siRNA1 achieved a better transfection efficiency, so we chose it for future experiments.

Under a scanning electron microscope (SEM), the swelling, the holes in the cell membrane, and the rupture of the cell surface were clearly visible in si-NC group, while the pyroptosis phenotypes were improved in NEK7 siRNA group (Fig. 2e). Consistent to cellular phenotype changes and PI and IF staining results, the protein levels of pyroptosis-related factors, including NLRP3 and GSDMD-N, were significantly decreased after NEK7 knockdown (Fig. 2f, g). At the same time, IL-1β content in the supernatant was significantly reduced after NEK7 knockdown (Fig. 2h), further indicate that NEK7 knockdown could abolish ATP + LPS-induced pyroptosis in vitro.

### Effect of NEK7 on chronic dextran sulfate sodium (DSS)-induced IBD model in mice in vitro

**Fig. 3:**
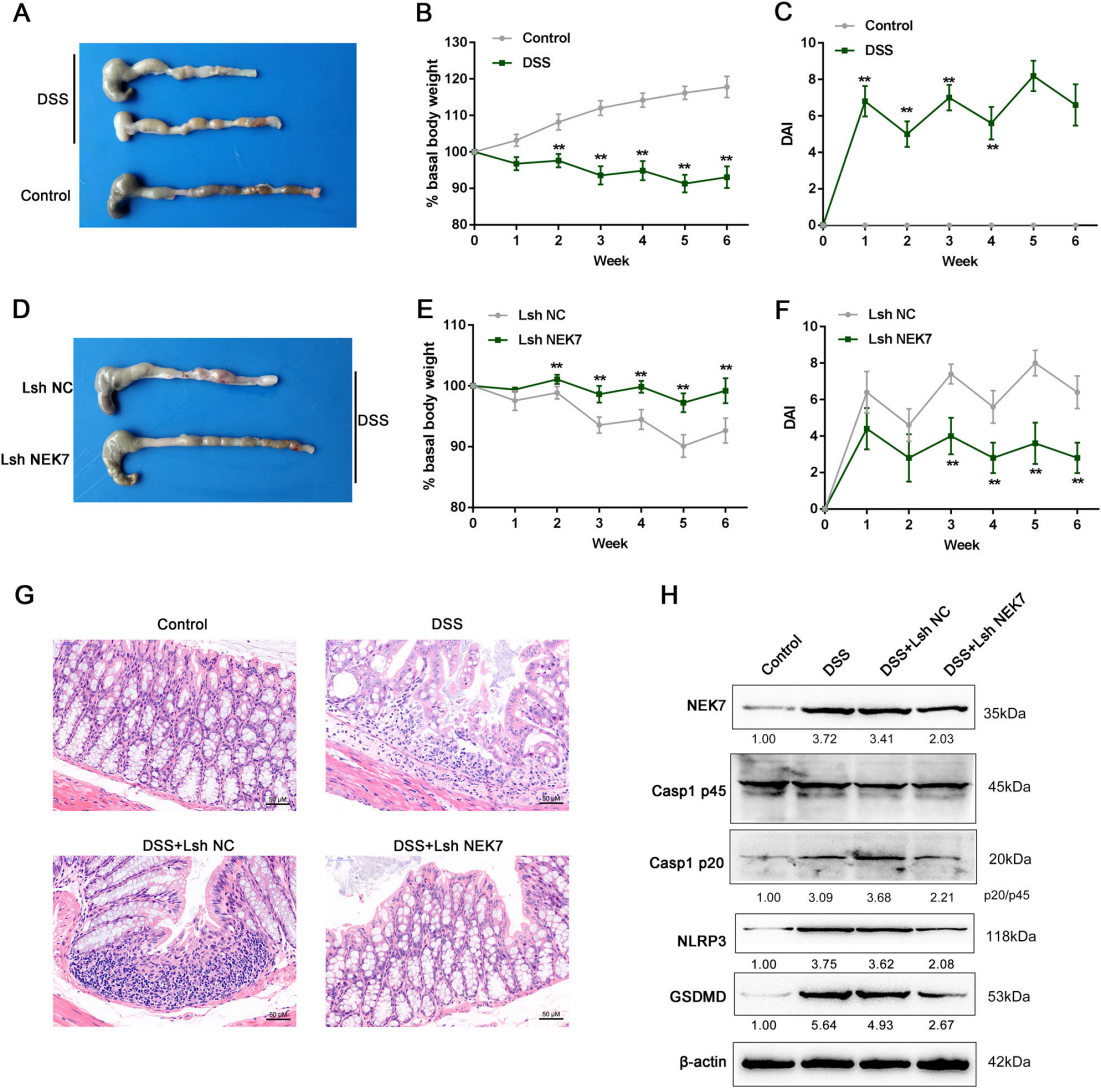
Effect of NEK7 on dextran sulfate sodium (DSS)-induced IBD mouse model in vitro. **a** Macroscopic changes in the colons of mice that received 4 cycles of DSS treatment. Representative image of colons from DSS administration (upper panels). Shortening of the colon length and thickening of the colonic wall were pronounced in DSS-treated mice. **b** Body weight and **c** DAI of mice in DSS treatment and control groups was determined once per 2 week from 0 to 6th week after DSS administration. Intraperitoneal injection of Lsh NEK7 were performed on mice (at week 0, 2, and 4) and **d** the macroscopic changes, **e** body weight, **f** DAI, and **g** pathological changes were examined and infiltration of inflammatory cells, mucosal necrosis, transmural inflammation, ulcerations and loss of cryptal cells were observed in the colon following induction of colitis with DSS treatment (scale bar, 50 μm). **h** The protein levels of NEK7, Caspase-1 (p45, p20), NLRP3, and GSDMD were examined by Immunoblotting. **P < 0.01, compared to control or Lsh NC group.

After confirming that NEK7 knockdown could abolish pyroptosis in MODE-K cells, next, we examined the effects of NEK7 knockdown via establishing DSS-induced IBD mouse model. As shown in Fig. 3a, DSS-treated mice showed serious inflammation, which was manifested as shorter, thicker and erythematous colons. DSS-treated mice suffered a significantly body weight loss (Fig. 3b); during the recovery phase (when the mice were fed with distilled water), the trend of weight loss in mice tended to be flat (Fig. 3b). Consistent with these changes, the DAI scores were sharply increased in DSS-treated mice (Fig. 3c); the trend of DAI score increase in mice tended to be attenuated during the recovery phase (Fig. 3c). Within macroscopic histological observations, DSS-treated mice presented an extensive bowel edema and epithelial cell destruction via large ulcers (Fig. 3g). These data indicate the success establishment of DSS-induced chronic IBD mouse model.

To investigate the effects of NEK7 knockdown in vivo, we performed intraperitoneal injection of Lsh-NEK7 on IBD mice. DSS‑treated NEK7-silenced mice exhibited minor erosions and mild edema within the colon (Fig. 3d). The body weight loss (Fig. 3e) and the increases in DAI scores (Fig. 3f) caused by DSS treatment was significantly rescued. Histological examination showed the typical histological characteristics of the colon and the small bowel in Lsh NC (negative control)-injected and Lsh NEK7-injected mice (Fig. 3g). After DSS administration, the colonic wall was significantly thickened, and a large number of inflammatory cells were infiltrated and accumulated through the wall. Moreover, upon DSS stimulation, we could clearly observe changes in the crypt structure and erosion of the epithelial surface within the colons of the Lsh NC-injected mice. Lsh NEK7-injected mice exhibited medium leukocyte infiltration, submucosal edema, and partial retention of the crypt structure and of epithelial surface (Fig. 3g). Consistently, Caspase-1 (p20) protein could be detected, the ratio of p20/p45, and the protein levels of NEK7, Caspase-1 (p45), NLRP3, and GSDMD were increased after DSS stimulation (Fig. 3h). After NEK7 knockdown, DSS-induced increases in p20/p45 and these proteins were reduced (Fig. 3h). These data indicate that NEK7 knockdown could improve DSS-induced chronic IBD in mice, possibly via affecting the pyroptosis in cells.

### LPS-induced activation of TLR4/NF-κB increase NEK7 expression by enhancing the transcriptional activity of NEK7

It has been reported that NEK7 could form a complex with NLRP3 in activated macrophages and interact with ASC oligomerization and ASC speckle formation that could be cleared without NEK7^21^. To investigate the mechanism of NEK7 modulating the pyroptosis in MODE-K cells and IBD in mice, we performed Co-IP and GST pull-down assays to confirm NEK7 could interact with NLRP3 here. We performed Co-IP assays to construct Flag-NEK7 and Myc-NLRP3 vectors and to co-transfect them into MODE-K cells. As confirmed by Western blotting, NEK7 protein interacts with NLRP3 protein in cells (Supplementary Fig. S2A). Upon GST pull-down assays, it has been revealed by Western blotting that GST-tagged NEK7 protein could pull-down His-tagged NLRP3 protein (Supplementary Fig. S2B), which suggests that there is a close interaction between NEK7 and NLRP3 proteins.

**Fig. 4:**
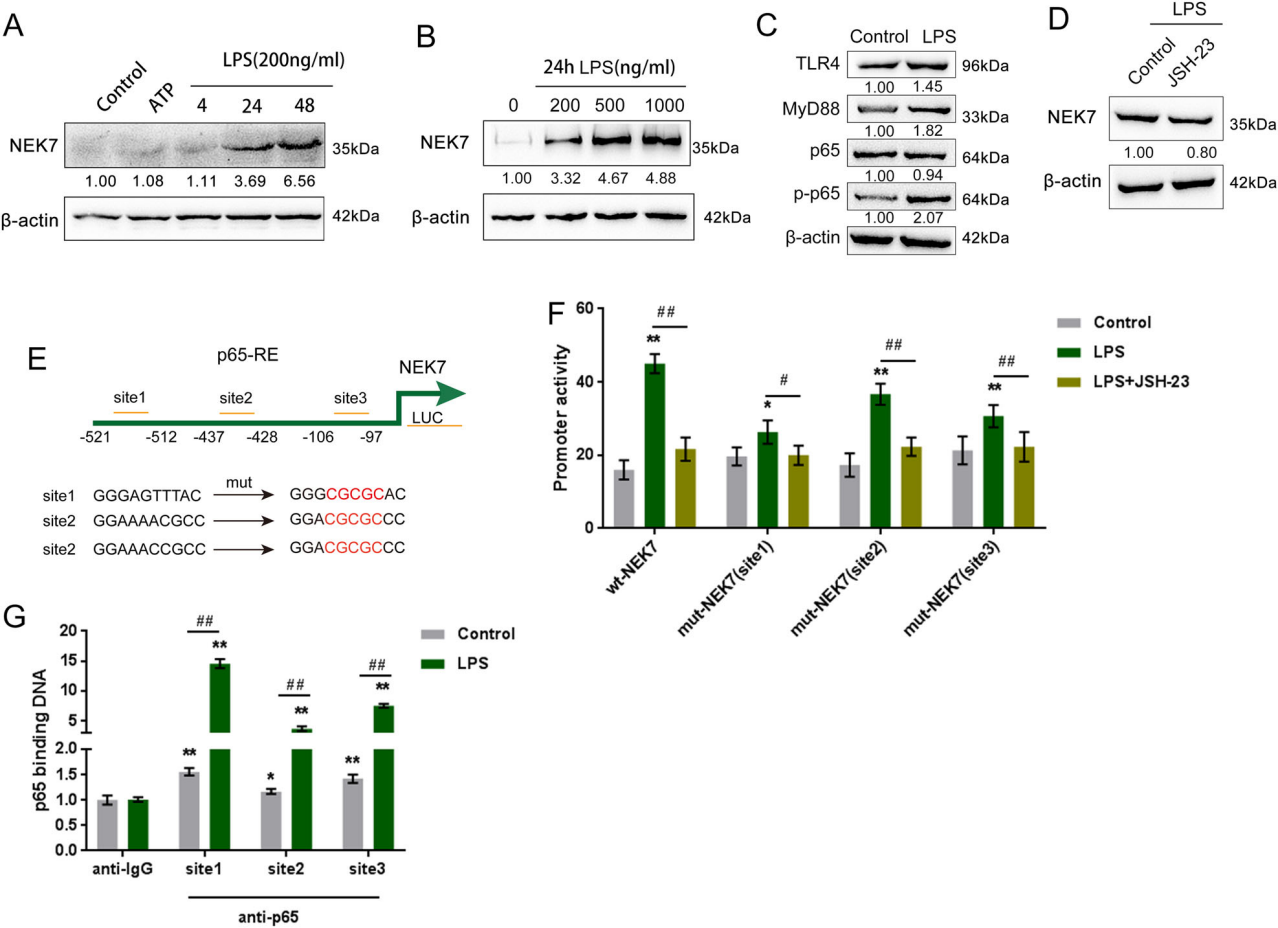
LPS-induced activation of TLR4/NF-κB increase NEK7 expression by enhancing the transcriptional activity of NEK7. **a** MODE-K cells were treated with ATP or LPS (200 ng/ml) for 4, 24, or 48 h and examined for the protein levels of NEK7. **b** MODE-K cells were treated with 0, 200, 500, and 1000 ng/ml LPS for 24 h and examined for the protein levels of NEK7. **c** MODE-K cells were treated with 200 ng/ml LPS for 24 h and examined for the protein levels of TLR4, MyD88, and p-p65. **d** MODE-K cells were treated with LPS and examined for the protein levels of NEK7 in the presence or absence of JSH-23. **e** A schematic diagram showing the predicted bindings between p65 response element (p65 RE) and NEK7 promoter region and the mutations in the predicted bindings. **f** These luciferase reporter vectors were transfected into MODE-K cells, treated with control, 200 ng/ml LPS, or 200 ng/ml LPS + 10 μM JSH-23 for 24 h, and examined for luciferase activity. **g** ChIP assays were performed with IgG or p65 antibodies to confirm the binding of p65 to NEK7. *P < 0.05, **P < 0.01, compared to control or anti-IgG group; ^#^*P* < 0.05, ^##^*P* < 0.01, compared to LPS or control group.

We have revealed that ATP + LPS stimulation could successfully induce the pyroptosis in MODE-K cells, which could be abolished by NEK7 silence; however, NEK7 protein levels were not significantly increased by short-term LPS alone stimulation (4 h) alone (Fig. 4a). To investigate the underlying mechanism, we examined the protein levels of NEK7 in response to ATP treatment alone, or 200 ng/ml LPS therapy for 4, 24, and 48 h. As shown in Fig. 4a, only 200 ng/ml LPS therapy for 24 and 48 h could remarkably upregulate NEK7 protein level, suggesting that NEK7 might be transcriptionally regulated upon chronic inflammation. Moreover, 200, 500, and 1000 ng/ml LPS treatment induced a dose-dependent increase in NEK7 protein levels (Fig. 4b). Mechanically, LPS is a strong activator of the nuclear factor-κB (NF-κB) pathway, which plays a key role in the inflammation response^28^; here, the protein levels of TLR4, MyD88, and p-p65 were examined under LPS stimulation. According to Fig. 4c, LPS stimulation could remarkably upregulate the protein levels of TLR4, MyD88, and p-p65. More importantly, under LPS stimulation, the treatment of JSH-23, an inhibitor of p65, caused a significant decrease in NEK7 protein level (Fig. 4d), suggesting that LPS might induce the upregulation of NEK7 via TLR4/NF-κB activation.

As reported by GTex online database, NEK7 and RELA expression was positively correlated in small intestine tissues (Supplementary Fig. S3A). Moreover, online chromatin immunoprecipitation data demonstrated that RELA binds upstream of NEK7 in multiple cells (Supplementary Fig. S3B). Thus, we hypothesize that RELA may activate the transcription of NEK7 via targeting its promoter region and performed luciferase reporter and ChIP assays to validate the hypothesis. Figure 4e shows the structures of NEK7 luciferase reporter vectors containing the predicted binding sites of p65 response element (p65 RE) or the mutation sites. We transfected MODE-K cells with these vectors, treated them with LPS alone or LPS + JSH-23, and examined them for the transcriptional activity. According to Fig. 4f, LPS remarkably upregulated the transcriptional activity of wt/mut NEK7 vectors; however, JSH-23 treatment could eliminate this effect. As a further confirmation, it has been reported by real-time ChIP assay that the level of p65 antibody targeting NEK7 promoter could be significantly increased, compared to that of IgG (Fig. 4g), which indicates that p65 might activate NEK7 expression via targeting its promoter upon any putative binding site.

## Discussion

Herein, a significant upregulation of NEK7 mRNA and protein expression and pyroptosis-associated factors, including Caspase-1 (p45, p20), NLRP3, and GSDMD, were observed in IBD tissue samples. In vitro inflammasome stimulation and cellular pyroptosis model and in vivo DSS-induced chronic colitis model was successfully conducted in MODE-K cells and mice, respectively. NEK7 knockdown abolish ATP + LPS-induced pyroptosis in vitro and improved DSS-induced chronic colitis in vivo. Regarding the underlying mechanism, NEK7 interacted with NLRP3, as revealed by Co-IP and GST pull-down assays, to exert its effects. Moreover, short-term (4 h) 200 ng/ml LPS treatment alone caused no significant changes in NEK7 protein level. TLR4/NF-κB signaling in MODE-K cells could be activated by LPS treatment. LPS-induced NEK7 upregulation could be significantly reversed by JSH-23, an inhibitor of p65. As revealed by LUC and ChIP assays, RELA might activate the transcription of NEK7 via targeting its promoter region.

Previous studies stated that NLRP3 inflammasome activation could be induced by several types of stimuli, including the intracellular potassium efflux in the potassium efflux model^29,30^, reactive oxygen species (ROS)^31^, the stimulatory substances like crystals or particles that enter cells^32^, and the metabolites like fatty acids, peptides, and toxins^33^. The inflammasome complex comprises various components, including NLRP3 and ASC, and cleaves and activates caspase-1, subsequently initiating pyroptosis^34,35^. Inflammasome signaling and pyroptosis are essential for the modulation of gastrointestinal health and disease, including IBD^13,36^. Consistent with previous studies, herein, we first observed a significant upregulation of the mRNA expression and protein levels of key factors of pyroptosis, including caspase-1 (p45), NLRP3, and GSDMD, in IBD tissue samples. Caspase-1 (p20) could only be detected in IBD tissues, indicating the cleavage of caspase-1 and the pyroptosis in IBD. In the meantime, NEK7 mRNA and protein expression could also be considerably upregulated in IBD tissues, indicating that NEK7 could exert a pyroptosis-associated effect on IBD.

As we have mentioned, it has been reported that NEK7 could not only exert an essential effect on intracellular potassium efflux, but also serve as a critical protein to inducing NLRP3 inflammasome activation. In addition, the absence of NEK7 could lead to specific blocking of NLRP3 inflammasome activation^21^. By using CRISPR/Cas9, it has been reported by Schmid-Burgk et al.^37^ that NEK7-knockout cells are less sensitive to nigericin-induced apoptosis, which could be mediated via low Caspase-1 and IL-1β expression. Herein, by stimulating mouse intestinal epithelial cell line, MODE-K, with ATP followed by LPS, we established an in vitro cell pyroptosis model. After knocking down NEK7 with transfection of NEK7 siRNA, the typical pyroptotic phenotypes in MODE-K cells apparently abolished: the numbers of the vesicle-like pyroptotic bodies and the ASC specks were significantly reduced. In the meantime, NEK7 knockdown significantly decreased the protein levels of NLRP3, caspase-1 (p20), and as GSDMD-N in MODE-K cells, as well as the release of IL-1β, indicating that NEK7 knockdown could inhibit NLRP3 inflammasome activation and subsequent pyroptosis in vitro.

Regarding its in vivo functions, NEK7-deficient mice with multiple sclerosis showed less IL-1β-related inflammatory disorders than wild-type mice^38^. During lung ischemia–reperfusion (IR), with NLPR3 being increased and induced, the activation of NLRP3 inflammasome could be suppressed after the inhibition of oxidative stress by the ROS scavenger edaravone. The preconditioning of Mcc950, an NLRP3 inhibitor, could inhibit the growth of proinflammatory cytokines IL-1β and IL-18 and disrupt the association between NLRP3 and NEK7 to remarkably reduce IR-caused lung injury^39^. Herein, we established a DSS-induced chronic colitis mouse model and conducted NEK7 knockdown by tail intravenous injection of Lsh NEK7. After NEK7 knockdown, the severe inflammation caused by DSS stimulation with the symtoms of shorter, thicker and erythematous colons was improved. DSS-induced body weight loss was rescued. In addition, not only DSS-induced extensive bowel edema, but also epithelial cell destruction by large ulcer in mice were largely improved. These observations indicate that NEK7 knockdown improved DSS-induced chronic colitis in mice. More importantly, along with the improvement of the inflammatory symptoms, DSS-induced upregulation of the protein levels of NEK7, NLRP3, caspase-1 (p20), and GSDMD-N were significantly decreased by NEK7 knockdown, indicating that NEK7 knockdown might affect DSS-induced chronic colitis in mice via modulating pyroptosis, possibly in an NLRP3-dependent manner.

Reportedly, NEK7 is required for the trigger of NLRP3 inflammasome. NEK7 binds to the NLRP3 leucine-rich repeat domain in a kinase-independent manner downstream from the induction of mitochondrial ROS^38^ or downstream of potassium efflux^21^. This interaction plays a critical role in NLRP3-ASC complex formation, ASC oligomerization, and caspase-1 activation. NEK7 promotes not only the NLRP3-dependent cellular inflammatory in response to the challenge of intraperitoneal monosodium urate, but also the progression of experimental autoimmune encephalitis in mice^38^. Herein, we also performed Co-IP and GST pull-down assays to confirm this interaction between NEK7 and NLRP3 in vitro and in vivo, further indicating that NEK7 affects the pyroptosis via interacting with NLRP3. Besides these stimuli, both the synergistic effect of TLR4/NF-κB signaling pathways and NLRP3 modification via ubiquitination exert a key effect on NLRP3 inflammasome activation as well^40^.

Interestingly, in MODE-K cells, although LPS therapy could upregulate NEK7 protein levels in a time-dependent manner, 200 ng/ml LPS therapy alone for short-term stimulation (4 h) caused no significant upregulation in NEK7 protein levels. However, the mRNA expression and protein levels of NEK7 were indeed higher in ulcerative colitis tissues. These findings indicate the existence of another mechanism by which NEK7 might be regulated upon chronic inflammation, most possibly a transcriptional regulation-related mechanism. Mechanically, LPS is a strong activator of the nuclear factor-κB (NF-κB) pathway, which plays a key role in the inflammation response^28^. In the canonical NF-κB pathway cascade^41^, the phosphorylation of IκB kinase (IKK) will lead to the phosphorylation of IκB, the cytoplasmic inhibitor of the NF-κB complex, a heterodimer composed of p50 and RelA/p65. The subsequent ubiquitination and proteasome-mediated degradation of IκB will then cause the release and nuclear translocation of the NF-κB complex. Once inside the nucleus, p65 engages the cognate κB enhancers and regulates the expression of downstream genes^41–43^. Here, LPS stimulation could remarkably upregulate the protein levels of TLR4, MyD88, and p-p65. More importantly, LPS-induced upregulation of NEK7 could be downregulated by JSH-23, a p65 inhibitor, suggesting that the expression of NEK7 was regulated by p65 in a transcriptional regulatory-manner. Afterward, LUC and ChIP assays both indicated that RELA could activate the transcription of NEK7 via targeting its promoter region, indicating that upon chronic inflammation, NEK7 expression is transcriptionally regulated by p65.

**Fig. 5:**
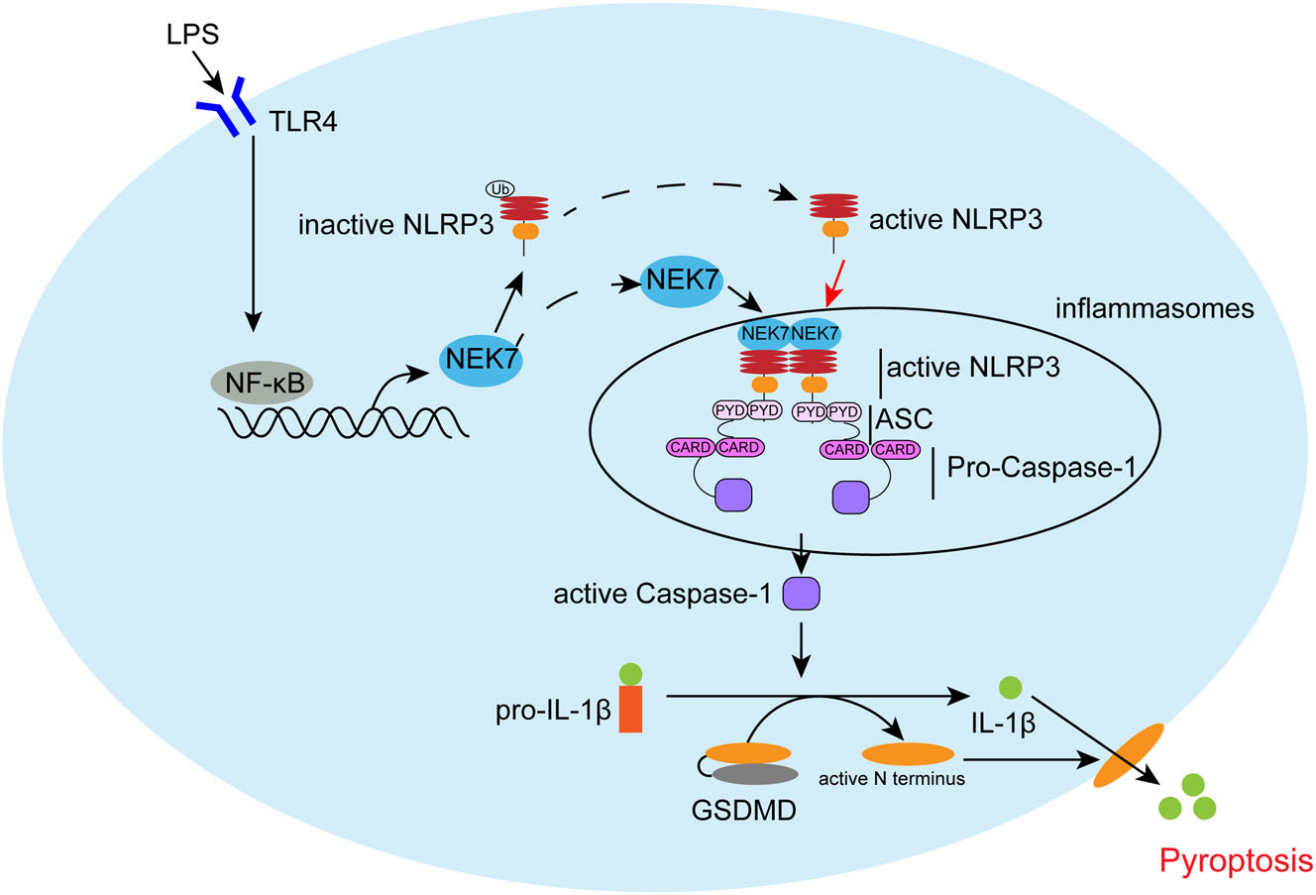
A mechanism diagram. LPS-induced TLR4/NF-κB activation causes an increase in NEK7 expression by RELA binding NEK7 promoter region. NEK7 interacts with NLRP3 to modulate NLRP3 inflammasome activation, therefore modulating the pyroptosis.

In summary, LPS-induced TLR4/NF-κB activation causes an increase in NEK7 expression by RELA binding NEK7 promoter region. NEK7 interacts with NLRP3 to modulate NLRP3 inflammasome activation, therefore modulating the pyroptosis in MODE-K cells and DSS-induced chronic colitis in mice (Fig. 5). We provide a novel mechanism of NEK7-NLRP3 interaction affecting IBD via pyroptosis.

## Supplementary information

table s1

fig.S1

fig.S2

fig.S3
