# Supplementary material for: NEK7 interacts with NLRP3 to modulate the pyroptosis in inflammatory bowel disease via NF-κB signaling
Source: Cell Death Dis. 2019 Dec 2;10(12):906. doi: 10.1038/s41419-019-2157-1 (PMC6885517; doi:10.1038/s41419-019-2157-1)
Supplement: Supplementary file 1 — table s1 [file 41419_2019_2157_MOESM1_ESM.docx]

| Antibody target genes | Cat. No. | Company | Country |
| --- | --- | --- | --- |
| NEK7 | [70-ab34287-050](http://www.liankebio.com/product-40987.html) | Multisciences Biotech, Co.ltd | Hangzhou ,China |
| Caspase-1 | [22915-1-AP](https://www.ptglab.com/products/CASP1-Antibody-22915-1-AP.htm) | proteintech | IL, USA |
| NLRP3 | 19771-1-AP | proteintech | IL, USA |
| GSDMD | ab209845 | abcam | Cambrige, UK |
| TLR4 | 19811-1-AP | proteintech | IL, USA |
| MyD88 | 23230-1-AP | proteintech | IL, USA |
| P65 | 10745-1-AP | proteintech | IL, USA |
| p-p65 | ab194726 | abcam | Cambrige, UK |
| β-actin | 60008-1-Ig | proteintech | IL, USA |
| Flag-tag | 20543-1-AP | proteintech | IL, USA |
| Myc-tag | 16286-1-AP | proteintech | IL, USA |
| GST-tag | 10000-0-AP | proteintech | IL, USA |
| Goat anti-Mouse IgG | GAM0072 | Multisciences Biotech, Co.ltd | Hangzhou ,China |
| Goat anti-Rabbit IgG | GAR0072 | Multisciences Biotech, Co.ltd | Hangzhou ,China |

Table S1 antibody information
