# Supplementary figures and images for: NEK7 interacts with NLRP3 to modulate the pyroptosis in inflammatory bowel disease via NF-κB signaling
Source: Cell Death Dis. 2019 Dec 2;10(12):906. doi: 10.1038/s41419-019-2157-1 (PMC6885517; doi:10.1038/s41419-019-2157-1)

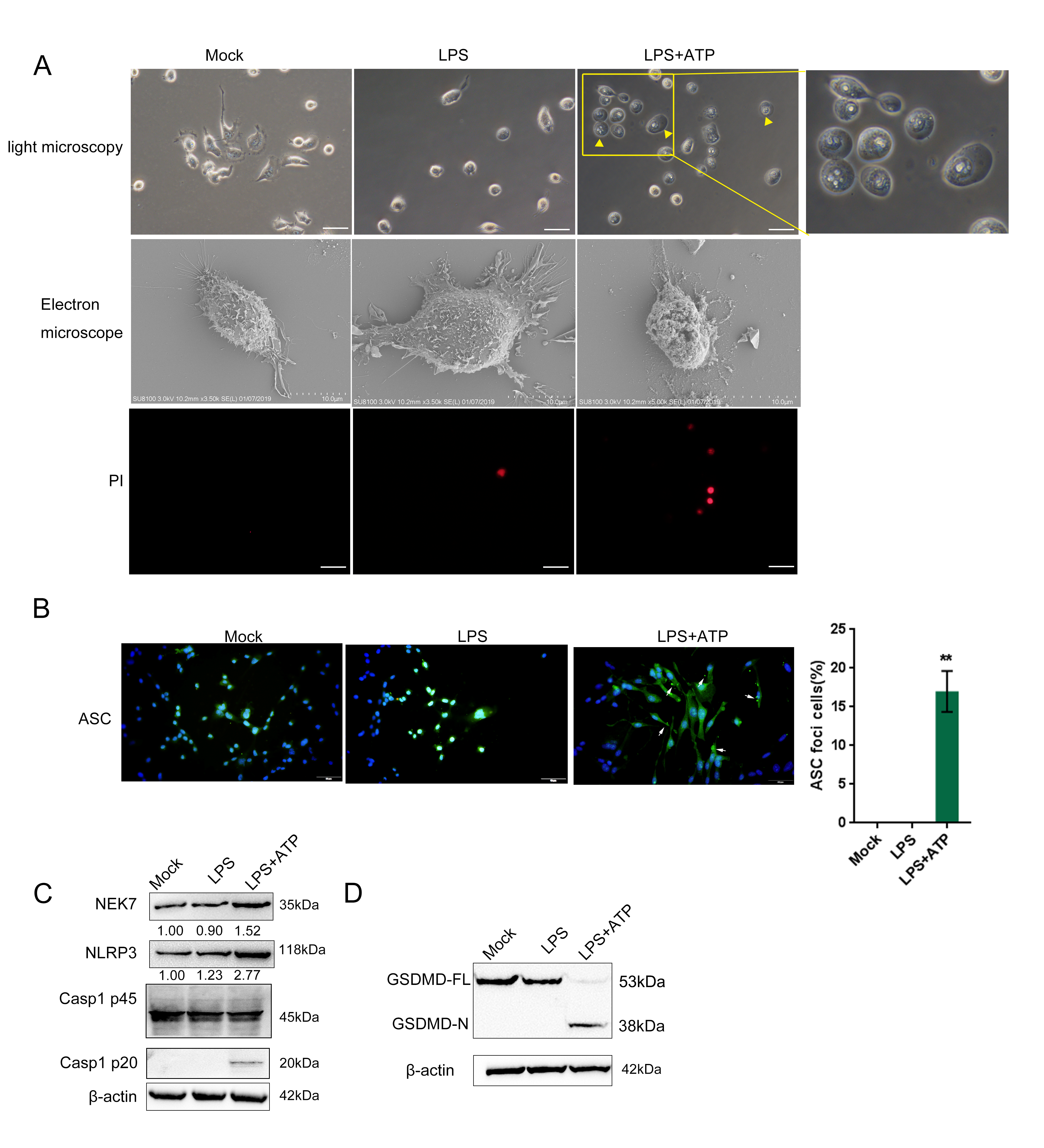

Supplement: Supplementary file 2 — fig.S1 [file 41419_2019_2157_MOESM2_ESM.tif]

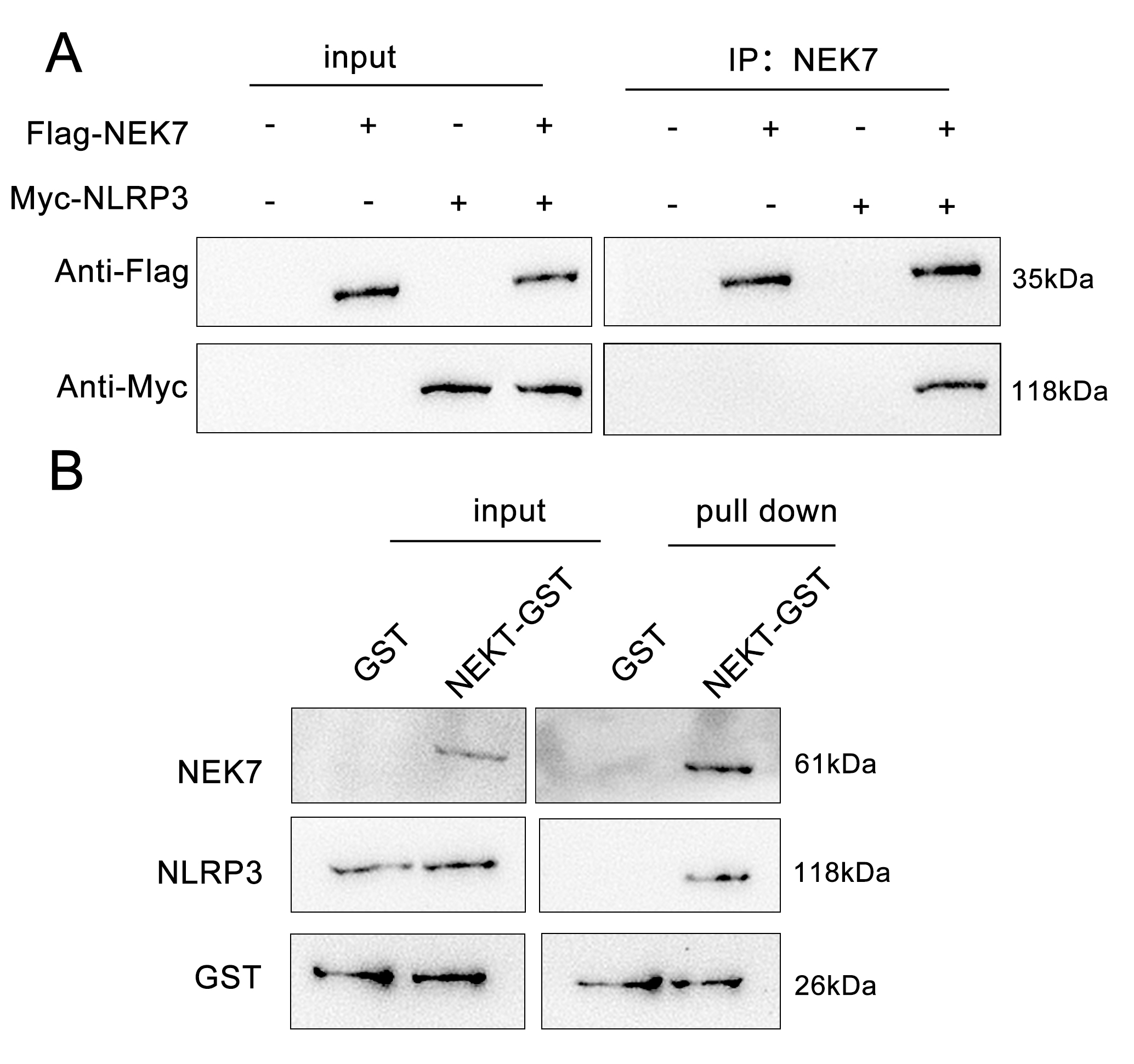

Supplement: Supplementary file 3 — fig.S2 [file 41419_2019_2157_MOESM3_ESM.tif]

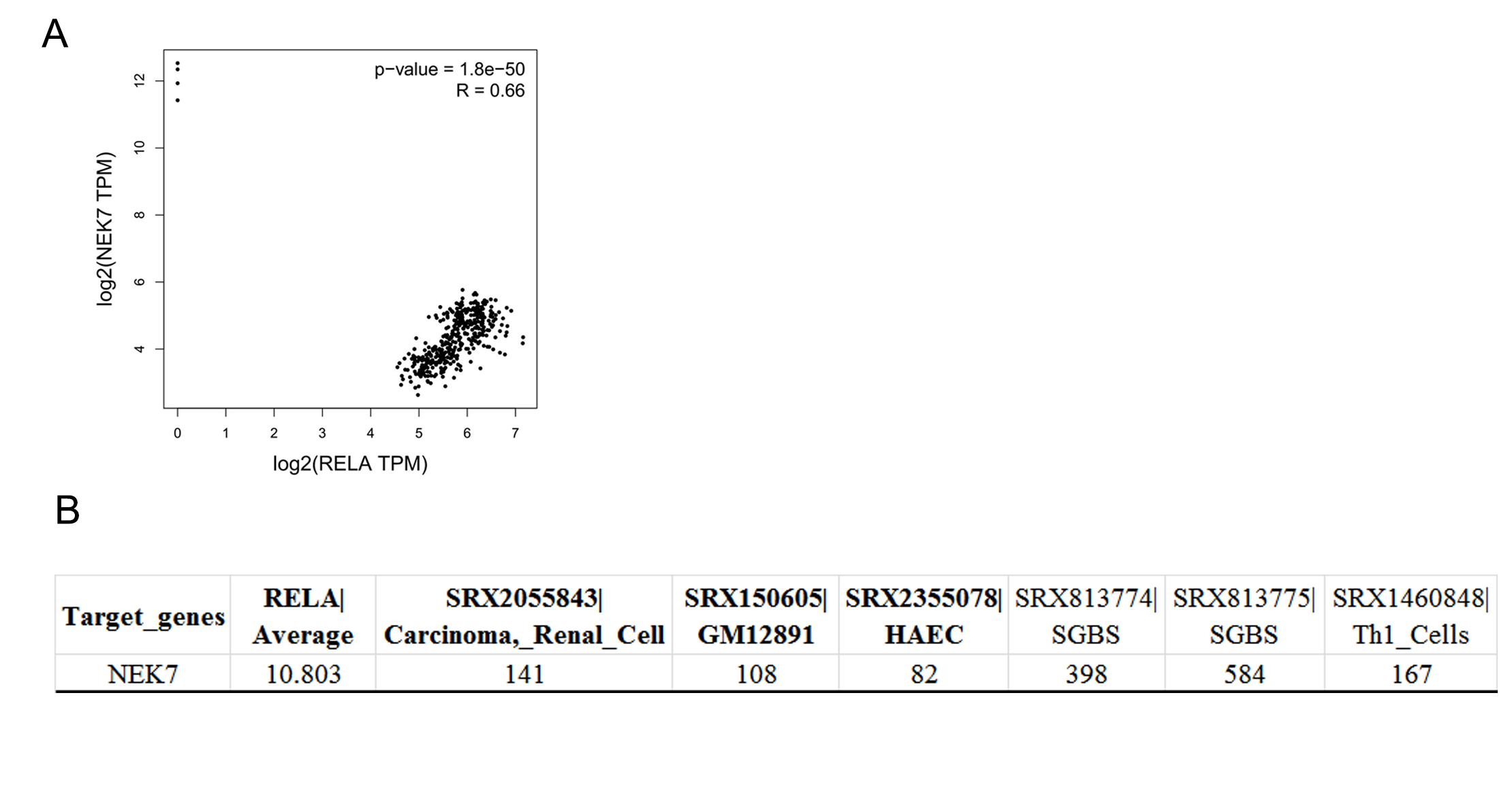

Supplement: Supplementary file 4 — fig.S3 [file 41419_2019_2157_MOESM4_ESM.tif]
